# Toddalolactone Protects Lipopolysaccharide-Induced Sepsis and Attenuates Lipopolysaccharide-Induced Inflammatory Response by Modulating HMGB1-NF-κB Translocation

**DOI:** 10.3389/fphar.2020.00109

**Published:** 2020-02-21

**Authors:** Jingyu Ni, Yuxuan Zhao, Jing Su, Zhihao Liu, Shiming Fang, Lan Li, Jie Deng, Guanwei Fan

**Affiliations:** ^1^ Tianjin Key Laboratory of Translational Research of TCM Prescription and Syndrome, First Teaching Hospital of Tianjin University of Traditional Chinese Medicine, Tianjin, China; ^2^ Tianjin State Key Laboratory of Modern Chinese Medicine, Tianjin University of Traditional Chinese Medicine, Tianjin, China

**Keywords:** toddalolactone, NF-kappa B pathway, HMGB1, inflammation, sepsis

## Abstract

Toddalolactone (TA-8) is a main compound isolated from *Toddalia asiatica* (L.) Lam., and its anti-inflammatory activity and anti-inflammatory mechanism are less studied. In the present study, we investigated the anti-inflammatory effects of TA-8. Our experimental results showed that TA-8 inhibited the production of pro-inflammatory cytokines by both lipopolysaccharide (LPS)-activated RAW 264.7 cells and septic mice. Moreover, TA-8 suppressed the NF-κB transcriptional activity, reduced the nuclear translocation and phosphorylation of NF-κB, blocked the translocation of HMGB1 from the nucleus to cytosol, and decreased LPS-induced up-regulation of TLR4 and IKBKB expression, and decreased IκBα phosphorylation. In addition, the administration of TA-8 decreased LPS-induced liver damage markers (AST and ALT), attenuated infiltration of inflammatory cells and tissue damage of lung, liver, and kidney, and improved survival in septic mice. Taken together, these results suggested that toddalolactone protects LPS-induced sepsis and attenuates LPS-induced inflammatory response by modulating HMGB1-NF-κB translocation. TA-8 could potentially be a novel anti-inflammatory and immunosuppressive drug candidate in the treatment of sepsis and septic shock.

## Introduction

Sepsis is a complex syndrome caused by a host-mediated inflammatory response during infection and is the leading cause of death in intensive care units. Sepsis is triggered by the invasion of pathogens and their toxins, and its development process has two phases: the first phase involves an overwhelming burst of pro-inflammatory cytokines, with an increase in the number of inflammatory mediators and inflammatory cells, which inhibit pathogens causing pathological damage. Lack of adequate immune response often leads to increased infection and ultimately death in patients with sepsis (Schorr et al., 2014; Seymour et al., 2016). In the second phase, the host’s immune response is partially dysregulated, resulting in an imbalance between pro-inflammatory and anti-inflammatory cytokine responses that ultimately lead to life-threatening tissue damage, multiple organ failure, and vascular dysfunction. These are precursors that lead to death from sepsis (Schulte et al., 2013). In recent years, despite the rapid progress of anti-inflammatory therapy and multi-organ function support technology, the mortality rate of sepsis is still up to 30 to 70%. Although researchers and caregivers have made great progress in the field of sepsis, it is still one of the diseases with high morbidity and high mortality and this disease reduces the quality of life of many survivors. It is usually treated by finding the primary lesion and using anti-inflammatory and antibacterial agents.

Inflammation is a complex biological process of tissue damage or infection by recruiting regulatory host immune cells. Pharmacotherapy of inflammation is a globally important and challenging goal. Lipopolysaccharide (LPS) is a common pathogen present in the outer membrane of Gram-negative bacteria (Fullerton and Gilroy, 2016). It can be recognized by the cell surface differentiation factor 2 (MD-2) and activates toll-like receptor 4 (TLR4). LPS can also be recognized by toll-like receptor 2 (TLR2) (Oeckinghaus et al., 2011). Toll-like receptors play a pivotal role in the human immune defense. Once TLR4 is activated by LPS, a variety of intracellular signaling pathways are altered. These cascaded signaling pathways mainly include mitogen-activated protein kinase signaling (MAPKs) and nuclear factor kB signaling pathway (NF-κB). Under normal conditions, NF-κB is present in the cytoplasm in an inactive form, and binds to the NF-κB inhibitor-associated regulatory protein IκB family to form NF-κB dimer. The IκB family has three members: IκBα, IκBβ, and IκBϵ. Phosphorylation of IκB is an important step in the activation of NF-κB, which dissociates the NF-κB dimer and releases free NF-κB. Phosphorylated IκB is then ubiquitinated to a molecule that is degraded by the proteasome complex and controls intracellular protein homeostasis by degradation. Phosphorylation of IκB is mediated by the IκB kinase (IKK) complex, which consists of at least three subunits, including IKBKA and IKBKB kinases and a regulatory IKBKC subunit (Napetschnig and Wu, 2013; Sun, 2017). The NF-kB signaling pathway plays an important role in the inflammatory process and is involved in the regulation of gene expression related to immune responses and cell survival, such as TNF-α, interleukin (IL)-1β, and IL-10.

High mobility group protein B1 (HMGB1) is a nuclear protein involved in a variety of transcriptional processes. HMGB1 stimulates and activates the immune system when it is released into the extracellular environment. HMGB1 is normally present in the nucleus and binds to DNA in the nucleus. It is transferred from the nucleus to the cytoplasm under inflammatory conditions and eventually secreted outside the cell. Four hours after LPS stimulated mononuclear-macrophages, inflammatory factors began to release HMGB1, and its release is later than most proinflammatory inflammatory factors (Huang et al., 2010). HMGB1 is an important late inflammatory mediator of endotoxin lethal effect. Active release of HMGB-1 activates NF-kB transcription factor through TLR-4 and stimulates inflammatory factor production. With the release of cytokines such as TNF-α and IL-1β, they further promote the active release of HMGB1 by cells, and continue to expand the effect of HMGB1. HMGB1 is closely related to the lethal effect of sepsis. The administration of HMGB1 antagonist in the late stage of animal sepsis model can improve the survival rate, and the intensity of inflammatory response in HMGB1 knockout mice is significantly reduced (Wang et al., 2001).

Toddaloctone is the root or root skin of the *Toddalia asiatica* L. of the genus *Rutacara*, which has the efficacy of dispersing blood stasis, stopping bleeding, and fixing pain. It is widely used in the treatment of stroke injury, rheumatic arthralgia, and sore and swelling. It is the traditional medicine in Guangxi, Guizhou, and Yunnan provinces. Toddalolactone (TA-8) is one of its components, modern pharmacological studies show that toddalolactone has anti-thrombotic and anti-fibrotic effects *in vitro* and *in vivo* (Yu Bo et al., 2017). But its anti-inflammatory activity and anti-inflammatory mechanism are less studied. Herein, we evaluate the anti-inflammatory activity of TA-8 and explore its potential mechanism by the *in vitro* model of LPS-stimulated RAW264.7 cells and mouse sepsis model induced by intraperitoneal injection of LPS.

## Materials and Methods

### RAW264.7 Murine Macrophage Culture

RAW264.7 murine macrophage was obtained from the cell bank of Institute of Basic Medical Sciences, Chinese Academy of Medical Sciences (Beijing, China). RAW264.7 cells were maintained in DMEM containing 10% FBS at 37°C in a moist atmosphere with 5% CO_2_ and 95% air. When the cell confluency reached 80%, cells were stimulated by LPS (1.0 μg/ml) in the presence or absence of toddalolactone (TA-8).

### Cell Viability Assay

MTT assay was used to detect the viability of the cell viability. RAW264.7 cells were seeded on 96-well plates with a density of 4 × 10^3^ cells/ml in 100 μl complete medium for 2 h. Subsequently, the cells were incubated with various concentrations of TA-8 in 37°C and 5% CO_2_ incubator for 16 h. 10 μl MTT was added into each well and incubated for 4 h in the dark, and then culture medium was removed with extra addition of 150 μl dimethyl sulfoxide (DMSO) to resolve the formazan. Finally, the optical density (OD) of the formazan of each well was measured with a microplate reader (Molecular Devices, USA) at 570 nm.

### Immunofluorescence

RAW264.7 cells were inoculated at a density of 4,000 cells/well in six-well plates and the cells were attached for 24 h. RAW264.7 cells were pretreatment with TA-8 (10^–5^ mol/L, 10^–7^ mol/L) for 40 min, and then were stimulated with LPS (1 μg/ml) for 2 h. Cells were rinsed in phosphate buffered saline containing 0.25% Tween20 (PBST) for 3×3 min. Cells were inclubated with normal serum block for 30 min. Then cells were inclubated with primary antibody for 1 h at room temperature. Cells were rinsed in PBST for 3×3 min and were inclubated with secondary antibody for 30 min at room temperature. Cells were counterstained with dihydrochloride (DAPI) for 10 min. The cells were photographed under an inverted fluorescence microscope.

### Survival Rate

Forty mice (half male and female) were randomly divided into a model group and TA-8 20 mg/kg group, sepsis was induced by intraperitoneal injection of 10 mg/kg LPS. Drug was administered three times at intervals of 8 h, control group was injected with the equal volume of normal saline. Survival rate experimental observation time is 5 days.

### Animals

C57BL/6N mice weighing 20–22g obtained from Beijing Vital River Laboratory Animal Technology Co., Ltd. (Beijing, China) and were housed individually under standard conditions (12-h light/dark cycles with a room temperature of 22–24°C). Male mice were randomly divided into a control group, model group, TA-8 20 mg/kg group and TA-8 10mg/kg group (n =12 per group). One hour after pre-administration, sepsis was induced by intraperitoneal injection of 10 mg/kg LPS for 20 h, model group was injected with equal volume of normal saline.

### Histological Investigation

The samples were removed and placed in 4% buffered formaldehyde, dehydrated, embedded in paraffin, and sectioned into 4-μm-thick sections. Hematoxylin and eosin staining were then performed.

### Serum Enzymology Test

The contents of procalcitonin (PCT), alanine aminotransferase (ALT), aspartate aminotransferase (AST), serum creatinine (SCR), and urea nitrogen (BUN) in the serum of mice were detected according to the manufacturer’s protocol by using a Fuji Dri-Chem 3500i Biochemistry Analyzer (Fujifilm Ltd, Japan) (Hung et al., 2017).

### Real-Time Quantitative PCR Detecting

Total RNA was extracted from the RAW264.7 murine macrophage and mouse liver using *TRIzol RNA* extraction kit (Invitrogen, Carlsbad, CA, USA) and the quality and concentration of the RNA were verified by spectrophotometry with a Minispine Nucleic Acid Analyzer (Thermo, USA). Complementary DNA (cDNA) was generated from 1 μg of total RNA in a 20-μl reaction volume using a PrimeScript™ RT Reagent Kit (Transgen Biotech, Beijing, China). The primer sequences of TNF-α, HMGB1, IL-1β, IL-1α, COX-2, VCAM-1, iNOS, RANTES, IL-10, IL-6, IP-10, and CCL2 were synthesized by Sangon Biotech Co., Ltd. (Shanghai, China) and listed in **Table 1**. They were quantified by real-time quantitative (q) reverse transcriptase polymerase chain reaction (RT-PCR) based on SYBR green labeling with a Light Cycler 480 real-time system from Roche, Co. Ltd. (Switzerland). 2^−△△Ct^ method was used to calculate the relative expression of genes, while GAPDH was regarded as reference gene to standardize the relative expression levels of target genes.

**Table 1:** Real-time (RT)-PCR primers.

Primer		Primer sequence
TNF-α	Forward	TTCTGTCTACTGAACTTCGGGGTGATCGGTCC
	Reverse	GTATGAGAYAGCAAATCGGATGACGGTGTGGG
HMGB1	Forward	AATAGGAAAAGGATATTGCT
	Reverse	GCGCTAGAACCAACTTATGA
IL-1β	Forward	GACCTTCCAGGATGAGGACA
	Reverse	AGCTCATATGGGTCCGACGC
IL-1α	Forward	CACCCGACTTTGTTCTTTGG
	Reverse	CCGACCTCATTTTCTTCTGG
COX-2	Forward	TGAGYACCGCAAACGCTTCTC
	Reverse	YGGACGAGGTTTTTCCACCAG
VCAM-1	Forward	GCTATGAGGATGGAAGACTCTGG
	Reverse	ACTTGTGCAGCCACCTGAGATC
GAPDH	Forward	ATGATTCTACCCACGGCAAG
	Reverse	CTGGAAGATGGTGATGGGTT
iNOS	Forward	GGCAGCCTGTGAGACCTTTG
	Reverse	GCATTGGAAGTGAAGCGTTTC
RANTES	Forward	ACACACTTGGCGGTTCCTT
	Reverse	CTGCTGCTTTGCCTACCTCT
IL-10	Forward	CCCTTTGCTATGGTGTCCTTTC
	Reverse	ATCTTCCTGGTTTCTCTTCCC
IL-6	Forward	TCCAGTTGCCTTCTTGGGAC
	Reverse	GTGTAATTAAGCCTCCGACTTG
IP-10	Forward	ATCCCTGCGAGCCTATCC
	Reverse	CATCCCAGCCACTTGAGC
CCL2	Forward	TGTGGAAAAGGTAGTGGATGC
	Reverse	TCTGTGCTGACCCCAAGAA

### Sodium Dodecyl Sulfate Polyacrylamide Gel Electrophoresis and Western Blot

The RAW264.7 murine macrophage and mouse liver tissues were homogenized in radioimmunoprecipitation assay (RIPA) lysis buffer and proteinase inhibitor cocktail (1:100; Beyotime, Haimen, China). For the isolation of nucleus and cytosol proteins, the nuclear protein isolation-translocation assay kit (Sangon Biotech Co., Ltd.) was employed according to the manufacturer’s protocol. The protein concentration was established using the BCA protein assay reagent according to the manufacturer’s instructions (Thermo Scientific, Waltham, MA, USA). Protein extracts were separated by electrophoresis using 4–15% SDS polyacrylamide gel and were electro-blotted onto 0.25 μm polyvinylidene difluoride (PVDF) membranes. Membranes were incubated with QuickBlock™ Western blocking solution (Beyotime, Haimen, China) for 15 min at room temperature, followed by incubation overnight with primary antibodies at 4 °C (1:1,000). Blots were washed five times with Tris-buffered saline/Tween 20 (TBST) and incubated with a 1:10,000 dilution of horseradish peroxidase conjugated secondary antibody for 2 h at room temperature. Blots were again washed five times with TBST and developed using an enhanced chemiluminescence (ECL) chemiluminescence substrate (Beyotime, Haimen, China). Band intensities were quantified using ImageJ software. Western blot analysis was performed with a specific primary antibody against NF-κB p65 (CST, USA), NF-κB p-p65 (CST, USA), TLR4(CST, USA), p-IKBα (CST, USA), IKBKB (CST, USA), HMGB1 (CST, USA), lamin B (CST, USA), β-actin (Proteintech). The results were expressed as the relative density to β-actin, glyceraldehyde 3-phosphate dehydrogenase (GAPDH), or lamin B and then normalized to the mean value of the control group.

### Plasmid Transfection

The pTk-Renilla plasmid was presented by Professor Yan Zhu from the American College of TUFTS. The pGL4.32 [luc2P/NF-κB-RE/Hygro] vector plasmid was presented by Professor Jiang Min from Nankai University. Plasmid was extracted using an endotoxin-free plasmid rapid extraction kit (Tiangen biotech, Beijing, China). The RAW264.7 murine macrophage were seeded in 24-well plates and cultured for 4 h. When the cell confluence rate reached 70–80%, 50 μl of a mixture of pTk-Renilla plasmid, and pGL4.32 plasmid was added to each well. Then 50 μl of lipofectamine TM2000 was added to each well for 5 min. The liposome solution and the pellet were mixed and allowed to stand for 25 min. Within this 25 min, each well was replaced with 400 μl/well of new complete medium. One hundred microliters of DNA and liposome mixture were added to each well, then cells were stimulated by LPS (1.0 μg/ml) in the presence or absence of toddalolactone. Dual-Luciferase Reporter Assay System from Promega was used to measure transcriptional activity.

### Enzyme-Linked Immunosorbent Assay

The cells were seeded into 24-well plates. After cultivation of 12 h, RAW264.7 cells were simultaneously treated with LPS (1.0 μg/ml) and toddalolactone (0.01, 0.1, 1,10 μmol/L) in culture medium for 24 h. Cell culture media was centrifuged at 2,000×g for 10 min to remove debris and collected 50 μl cell-free supernatants to assay immediately. Pro-inflammatory cytokines including IL-1β and TNF-α of RAW264.7 murine macrophage was detected using ELISA Kit for IL-1β and TNF-α (Wuhan USCN business Co., Ltd).

### Statistical Analysis

The assays were performed three times. The experimental data are expressed as means ± SD. For survival, Kaplan-Meier plots were used and assessed using a logrank test. Statistical analysis was performed by one-way ANOVA of repeated experiments followed by Bonferroni’s multiple group comparison with Prism 5 (GraphPad). A p value < 0.05 was considered statistically significant.

## Results

### TA-8 Inhibited Lipopolysaccharide-Induced Pro-Inflammatory Cytokines and Messenger RNA Expression in RAW264.7 Cells

**Figure 1:**
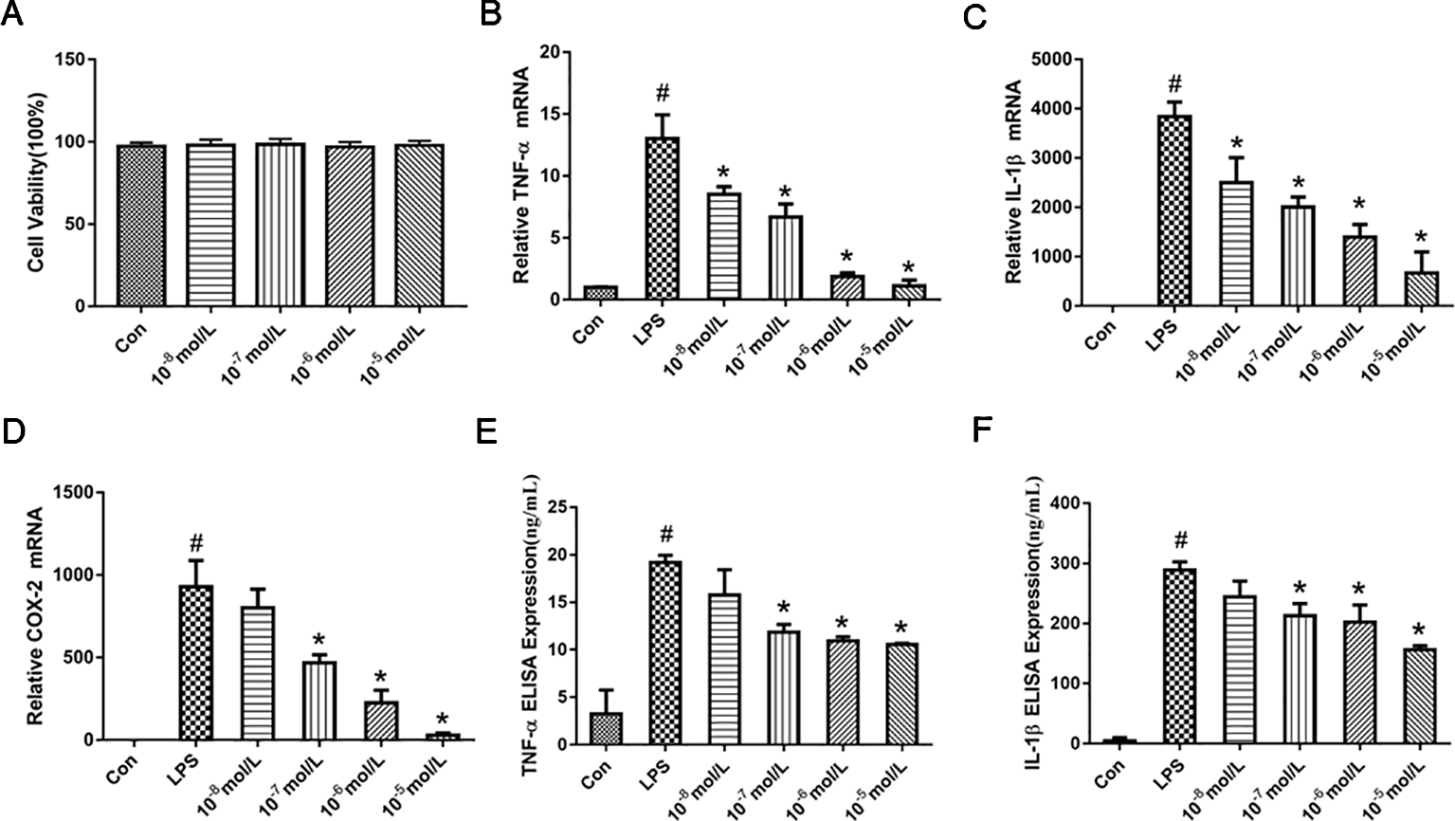
TA-8 inhibited lipopolysaccharide (LPS)-induced pro-inflammatory cytokines and messenger RNA (mRNA) expression in RAW264.7 cells. RAW264.7 cells were inoculated at a density of 4,000 cells/well in 96-well plates and divided into control group and drug concentration groups of different concentrations. After the cells were attached for 2 h, the drug concentration was 10^−5^, 10^−6^, 10^−7^, and 10^−8^ mol/L, respectively. After 16 h, the cell viability was detected by MTT assay. TA-8 had no significant effect on RAW264.7 cell viability (panel **A**). Total RNA was prepared for quantitative real-time (qRT)-PCR analysis of interleukin (IL)-6 and TNFα in cells stimulated with LPS (1 μg/ml) for 3.5 h in presence or absence of TA-8****. The mRNA levels of TNF-α (panel **B**), IL-1β (panel **C**), and COX2 (panel **D**) were determined using gene specific primers. The results are expressed as the ratio of optimal density to GAPDH. RAW264.7 was seeded in 24-well plates at a cell density of 10,000 cells/well, after pre-administration for 40 min, the model group and the drug-administered group were added with 1 μg/ml LPS. After 16 h of culture, the contents of TNF-α (panel **E**) and IL-1β (panel **F**) in the supernatant of the cells were determined using an ELISA kit. ^#^ *P* < 0.05 *versus* the control group; ^*^ *P* < 0.05, *versus* LPS-stimulated cells.

MTT assay was performed to exclude the possibility that the inhibitory effects were due to the cytotoxicity of TA-8. As shown in the **Figure 1A**, RAW264.7 cells viability has no significant difference after the cells were treated with TA-8. The results indicated that the influences of TA-8 on RAW264.7 cells were not due to the cytotoxicity of TA-8 and LPS. Release of cytokines is an important step in regulating host immune responses to inflammation. As shown in the **Figure 1**, TA-8 significantly suppressed the messenger RNA (mRNA) expression of TNF-α (**Figure 1B**), IL-1β (**Figure 1C**), and COX2 (**Figure 1D**) in the LPS-treated macrophage with a dose-dependent manner (*P* < 0.01). TA-8 significantly reduce the release of TNF-α (**Figure 1E**) and IL-1β (**Figure 1F**) in the LPS-treated macrophage (*P* < 0.01). These results illustrate its anti-inflammatory effects, as well as possible clinical effects.

### TA-8 Inhibited Lipopolysaccharide-Induced NF-κB Activation in RAW264.7 Cells

**Figure 2:**
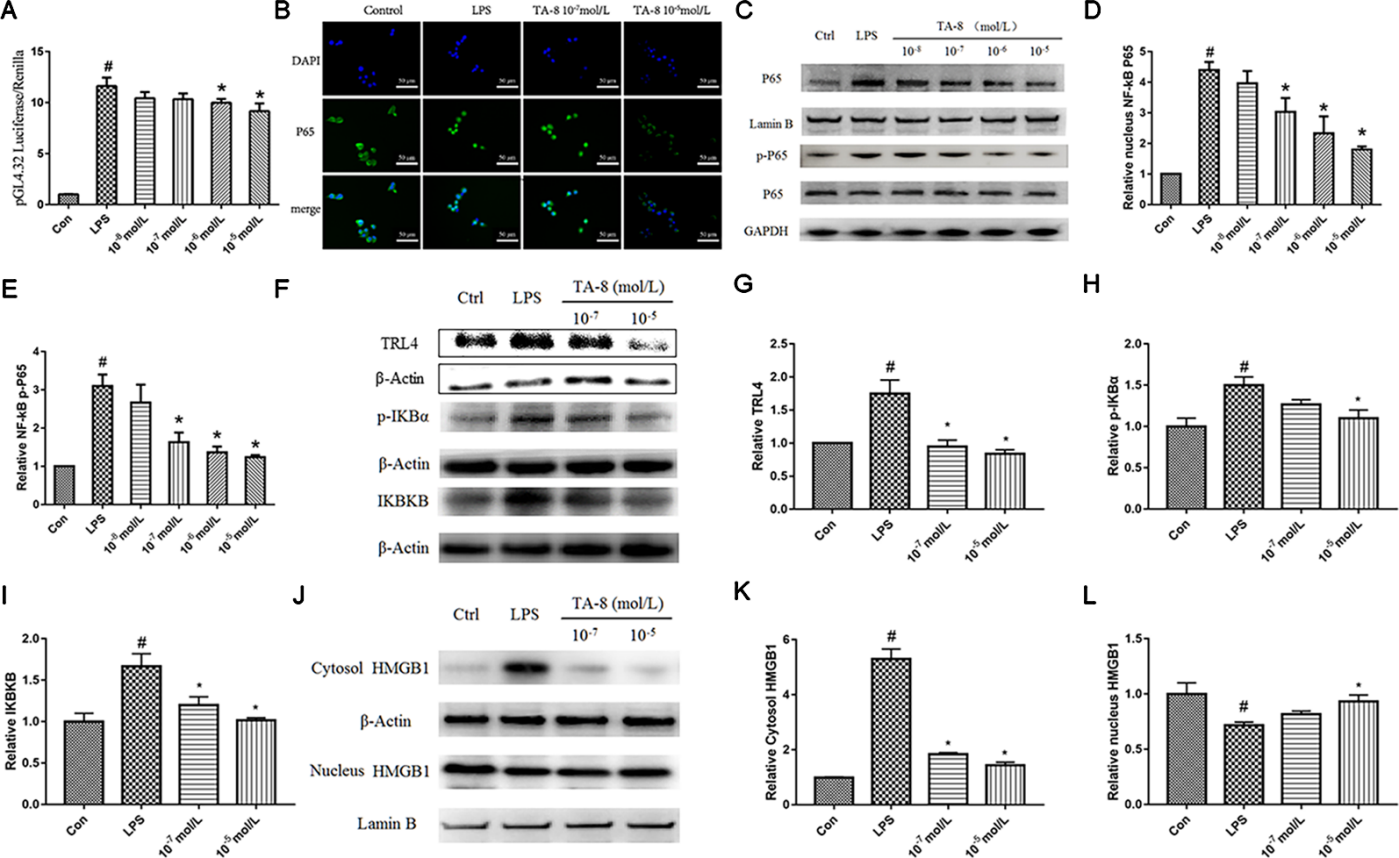
TA-8 inhibited lipopolysaccharide (LPS)-induced HMGB1-NF-κB translocation in RAW264.7 cells. **(A)** TA-8 can inhibit NF-κB transcriptional activity; **(B–E)** TA-8 can inhibit nuclear translocation and phosphorylation of NF-κB P65, RAW264.7 cells were pretreatment with TA-8 (10^−5^, 10^−6^, 10^−7^, and 10^−8^ mol/L) for 40 min, and then were stimulated with LPS (1 μg/ml) for 45 min. The nuclear transfer of p65 was detected by using inverted fluorescence microscopy. The level of P65 (nucleus), P65, p-P65, and GAPDH were examined using a specific antibody. **(F–I)** TA-8 can inhibit LPS-induced IκBα phosphorylation and up-regulation of TLR4-IKBKB expression. **(J–L)** TA-8 can significantly inhibit the decrease of HMGB1 content induced by LPS and inhibit the secretion of HMGB1 into cytoplasm. ^#^ *P* < 0.05 *versus* the control group; ^*^ *P* < 0.05, *versus* LPS-stimulated cells.

NF-κB is critically required for the LPS-induced transcriptional regulation of inflammation. We hypothesized that TA-8 is also highly likely to exert anti-inflammatory activity through the NF-κB signaling pathway. Therefore, we used NF-κB commercial plasmid transfection experiments and NF-κB. Western blot analysis experiments, the effects of NF-κB transcriptional activity and nuclear translocation after phosphorylation were examined, respectively. As a result, we found that TA-8 can inhibit NF-κB transcriptional activity (**Figure 2A**). To further investigate the effect of TA-8 on NF-κB transcription factors, we used inverted fluorescence microscopy to detect the nuclear transfer of p65 and examined the expression of NF-κB protein in the nucleus by using the western blot. As shown in **Figures 2B–D**, it was found that TA-8 can inhibit nuclear translocation of NF-κB. At the same time, the effect of TA-8 on the phosphorylation of NF-κB P65 protein was detected by western blot. We found that TA-8 inhibits phosphorylation of NF-κB P65 protein (**Figures 2D, E**). Based on the above results, we selected two optimal TA-8 drug concentrations to further investigate the effect of TA-8 on the upstream signaling pathway of NF-κB. It was found that TA-8 can inhibit LPS-induced IκBα phosphorylation and up-regulation of TRL4-IKBKB expression (**Figures 2F–I**).

### TA-8 Attenuates Lipopolysaccharide-Induced Inflammatory Response by Modulating HMGB1-NF-κB Translocation

HMGB1, also known as HMG1 or amphoterin, is released upon cell necrosis, apoptosis, and pyroptosis. It acts as a multifunctional alarmin that stimulates inflammation upon sterile or infectious insult. HMGB1 is both a nuclear protein and a secretory inflammatory factor. In the inflammatory state, it undergoes a positional shift from the nucleus to the cytoplasm to the extracellular fluid. In this study, nucleoprotein and plasma proteins of RAW264.7 cells under normal conditions and inflammatory conditions were extracted, and the positional changes of HMGB1 were detected. It can be seen from **Figures 2J–L** that TA-8 can significantly inhibit the decrease of HMGB1 content induced by LPS and inhibit the secretion of HMGB1 into cytoplasm.

### TA-8 Improves Survival in Septic Mice and Alleviates Lung, Liver, and Kidney Injury

**Figure 3:**
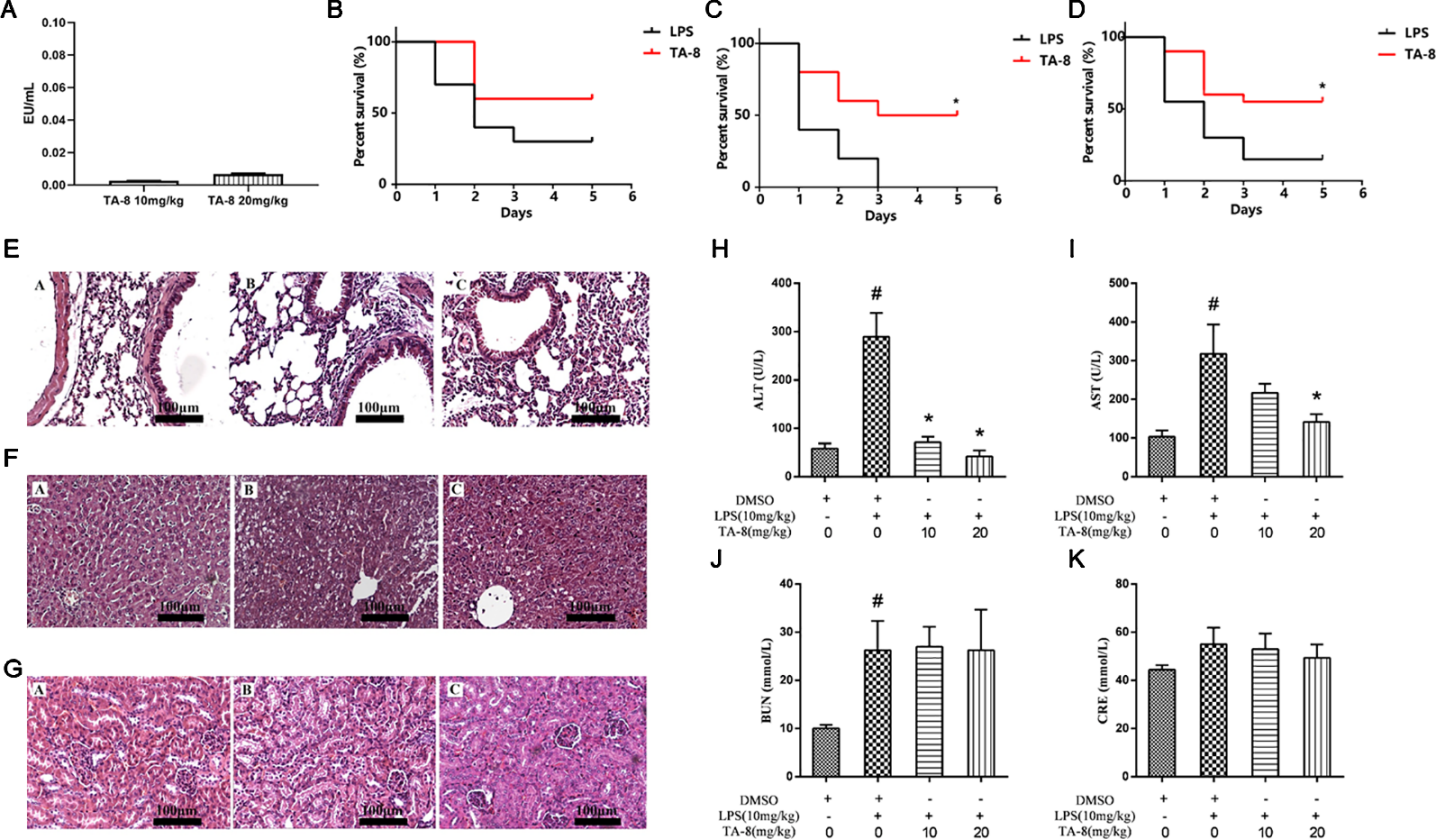
TA-8 effectively protects mice from lipopolysaccharide (LPS)-induced mortality and reduces lung, liver, and kidney injuries. We examined whether the drug was contaminated with endotoxin. Endotoxin residues have been detected by using limulus amebocyte lysate (LAL) test. The results showed that the endotoxin residue was less than 0.01 EU/ml, which was in compliance with the requirements for injectables (panel **A**). Ten male mice and ten female mice per group were treated with vehicle only or TA-8 (i.p.) after LPS injection (20 mg/kg, i.p.). Mice were monitored every 8 h for 5 days. Mice treated with TA-8 had significantly higher survival than model mice (female mice panel **B**; male mice panel **C**; all mice panel **D**). C57BL/6 mice were injected with TA-8 or vehicle [dimethyl sulfoxide (DMSO)] 1 h before LPS injection (20 mg/kg, i.p.), tissues of lung, liver and kidney were harvested 20 h after LPS injection. The results show H&E-staining of lung (panel **E**), liver (panel **F**), or kidney tissue (panel **G**) sections from the indicated group (×200). The figure is a representative of three independent experiments. C57BL/6 mice were injected with TA-8 or vehicle (DMSO) 1 h before LPS injection (10 mg/kg, i.p.), and the blood samples were harvested 20 h after LPS injection. The levels of ALT (panel **H**), AST (panel **I**), BUN (panel **J**), and CRE (panel **K**) were measured (n=3). ^#^ *P* < 0.05 *versus* the control group; ^*^ *P* < 0.05, *versus* LPS-stimulated mice.

First we examined whether the drug was contaminated with endotoxin. Endotoxin residues have been detected by using limulus amebocyte lysate (LAL) test. The results showed that the endotoxin residue was less than 0.01 EU/ml, which was in compliance with the requirements for injectables (**Figure 3A**). Survival was evaluated using Kaplan-Meier survival curves. As shown in **Figures 3B, D**, a decline in the survival rate was found in all model groups. Mice treated with TA-8 had significantly higher survival than model mice [male mice (**Figure 3B**), *p *< 0.05; female mice (**Figure 3C**), n.s; all mice (**Figure 3D**), *p *< 0.05].

To determine the effects of TA-8 on LPS-induced injury, we evaluated the histology of lung (**Figure 3E**), liver (**Figure 3F**), and kidney (**Figure 3G**) specimens stained with hematoxylin and eosin. In LPS-induced lung injury, the thickness of alveolar wall was increased, and the number of pulmonary alveolus was reduced by LPS injection. The administration of TA-8 repressed the swelling of alveolar wall and declined the number of pulmonary alveolus in LPS-challenged mice. Compared with the blank control group, the hepatic lobular structure of the liver tissue section of the model group was destroyed, and the structures of hepatic cord, boundary plate, and hepatic sinus were unrecognizable, and large-area hepatocytes showed balloon-like changes and hepatocytes necrosis into pieces. After TA-8 administration, the overall liver lesion was lighter than the model group. Compared with the blank control group, the renal tissue of the model group did not show obvious substantial lesions, only the changes of renal function were observed. Compared with the model group, a small part of the renal tubules had proteinuria in the kidney tissue section and rare tubeuria occurred after TA-8 administration.

To further study the effect of TA-8 on liver and kidney functions, serum ALT, AST, BUN, and CRE were detected by an automatic biochemical analyzer. ALT and AST are mainly distributed in liver cells, and ALT and AST are released into the blood when the liver is damaged. TA-8 can significantly reduce the content of ALT (**Figure 3H**) and AST (**Figure 3I**) in sepsis model mice, indicating that it can reduce liver cell damage and protect the dirty. BUN and CRE are commonly used indicators of renal function evaluation, and TA-8 has no significant effect on the increase in BUN (**Figure 3J**) and CRE (**Figure 3K**) levels caused by LPS.

### TA-8 Effectively Inhibited the Expression of Inflammatory Factors in Septic Mice

**Figure 4:**
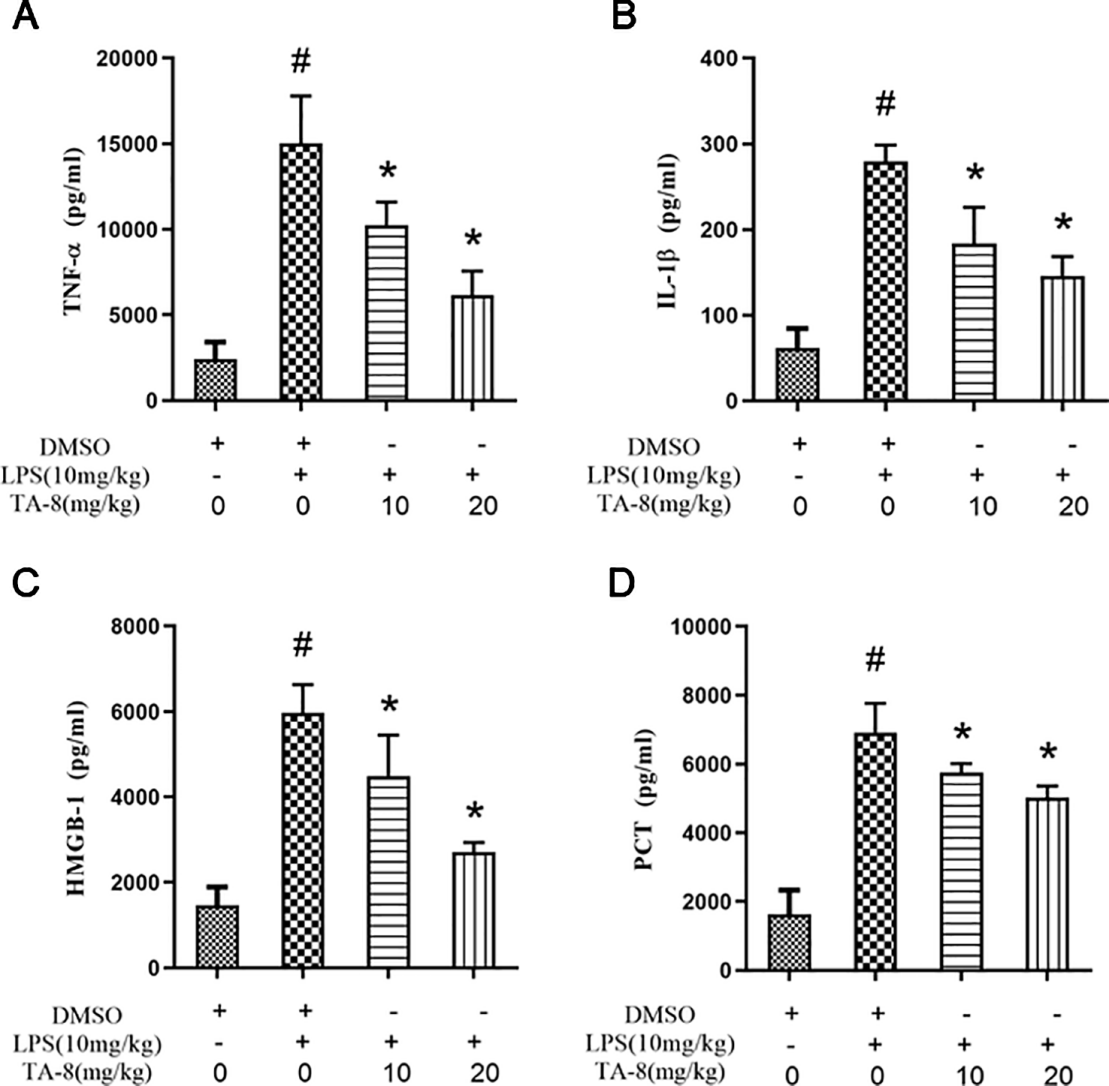
TA-8 effectively inhibited the expression of inflammatory factors in septic mice. Ten mice per group were treated with vehicle only or TA-8 (10 or 20 mg/kg, i.p.) 1 h before lipopolysaccharide (LPS) injection (10 mg/kg, i.p.). Serum samples were obtained from each mouse 4 or 16 h later. Amount of TNF-α (panel **A**), IL-1β (panel **B**), HMGB-1 (panel **C**), and PCT (panel **D**) in the serum were determined using the ELISA analysis. ^#^ *P* < 0.05 *versus* the control group; ^*^ *P* < 0.05, *versus* LPS-stimulated mice.

**Figure 5:**
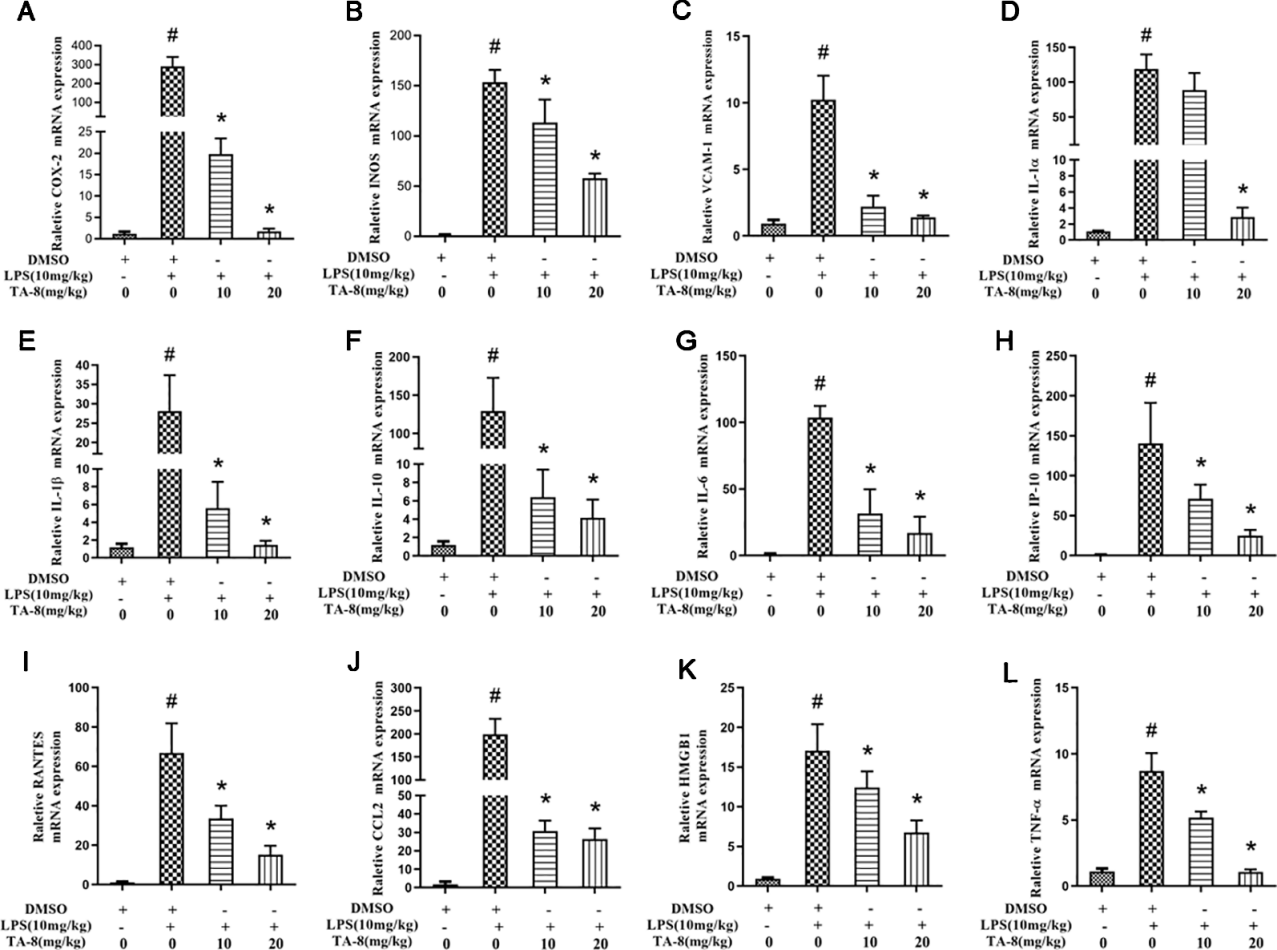
TA-8 significantly inhibited the messenger RNA (mRNA) expression of inflammatory factors in liver tissue during the period of multi-organ dysfunction syndrome (MODS) in sepsis. Mice were treated with vehicle only or TA-8 (10 or 20 mg/kg, i.p.) 1 h before lipopolysaccharide (LPS) injection (10 mg/kg, i.p.). Liver tissue samples were obtained from each mouse 20 h later. The mRNA levels of COX-2 (panel **A**), iNOS (panel **B**), VCAM (panel **C**), interleukin (IL)-1α (panel **D**), IL-1β (panel **E**), IL-10 (panel **F**), IL-6 (panel **G**), IP-10 (panel **H**), RANTES (panel **I**), CCL2 (panel **J**), HMGB1 (panel **K**), and TNF-α (panel **L**) were determined by quantitative real-time (qRT)-PCR. ^#^ *P* < 0.05 *versus* the control group; ^*^ *P* < 0.05, *versus* LPS-stimulated mice.

In this experiment, a mouse model of sepsis was induced by intraperitoneal injection of 10 mg/kg LPS after 1 h of pre-administration. We found that the high and low dose groups of TA-8 significantly inhibited the secretion of inflammatory factors TNF-α (**Figure 4A**) and IL-1β (**Figure 4B**) during the period of multi-organ dysfunction syndrome (MODS) in septic mice. HMGB1 is an important late inflammatory mediator and is closely related to the lethal effect of LPS. TA-8 can inhibit the expression of HMGB1 (**Figure 4C**) during the period of MODS. Procalcitonin (PCT) and C-reactive protein (CRP) play a special role in infectious diseases, the level of PCT and CRP protein in the infected body was significantly increased, and TA-8 significantly inhibited serum PCT (**Figure 4D**) levels during the period of MODS. At the same time, we found that TA-8 significantly inhibited the mRNA expression of COX2 (**Figure 5A**), iNOS (**Figure 5B**), VCAM (**Figure 5C**), IL-1 (**Figure 5D**), IL-1 (**Figure 5E**), IL-10 (**Figure 5F**), IL-6 (**Figure 5G**), IP-10 (**Figure 5H**), RANTES (**Figure 5I**), CCL2 (**Figure 5J**), HMGB1 (**Figure 5K**), and TNF-α (**Figure 5L**) in liver tissue during the period of MODS in sepsis. These results can demonstrate to some extent the inhibitory effect of TA-8 on the expression of organ inflammatory factors.

### TA-8 Effectively Attenuates Lipopolysaccharide-Induced HMGB1-NF-κB Translocation in Liver Tissue

**Figure 6:**
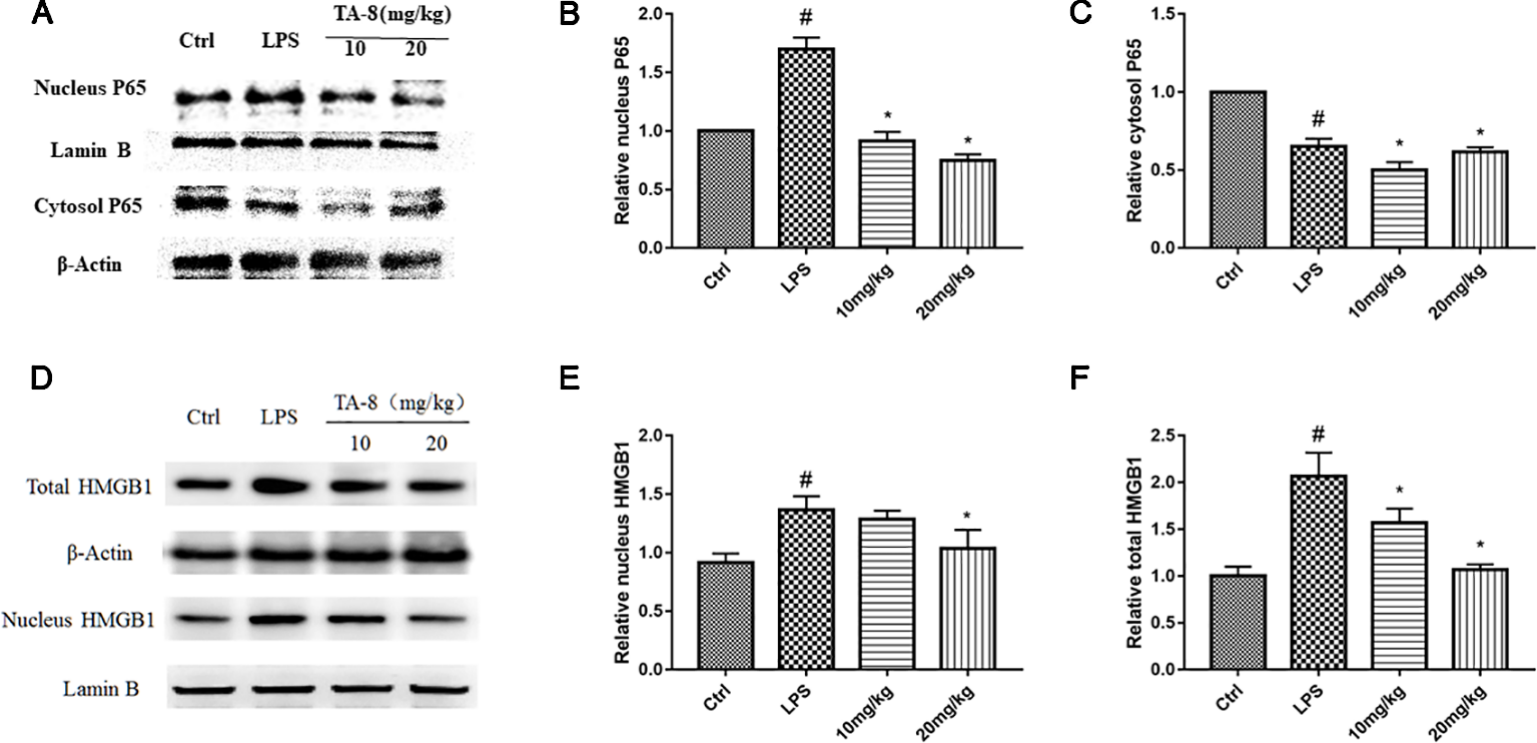
TA-8 effectively attenuates lipopolysaccharide (LPS)-induced HMGB1-NF-κB translocation in liver tissue. Mice were treated with vehicle only or TA-8 (10 or 20 mg/kg, i.p.) 1 h before LPS injection (10 mg/kg, i.p.). Liver tissue samples were obtained from each mouse 20 h later. The level of P65 (nucleus and cytosol) and HMGB1 (nucleus and total) were examined using a specific antibody. **(A–C)** TA-8 can inhibit the nuclear transfer of NF-κB P65 in LPS-induced sepsis in C57BL/6 mice; **(D–F)** TA-8 significantly reduces nuclear and total HMGB1 levels. The experiments were repeated three times, respectively, and similar results were obtained. ^#^ *P* < 0.05 versus the control group; *P < 0.05 versus LPS stimulated mice.

It was revealed from the cell experiments that the expression levels of HMGB1 were decreased in the nucleus and increased in the cytoplasm after induction by LPS. Compared with the RAW264.7 cell inflammation model, the animal inflammation model is more complicated. After 20 h of LPS stimulation, we found that TA-8 can inhibit the nuclear transfer of NF-κB P65 in LPS-induced sepsis in C57BL/6 mice (**Figures 6A–C**). After LPS stimulation, HMGB1 significantly increased in the nucleus, and the total protein of HMGB1 was also higher than that of the control group. TA-8 significantly reduced nuclear and total HMGB1 levels (**Figures 6D–F**).

**Figure 7:**
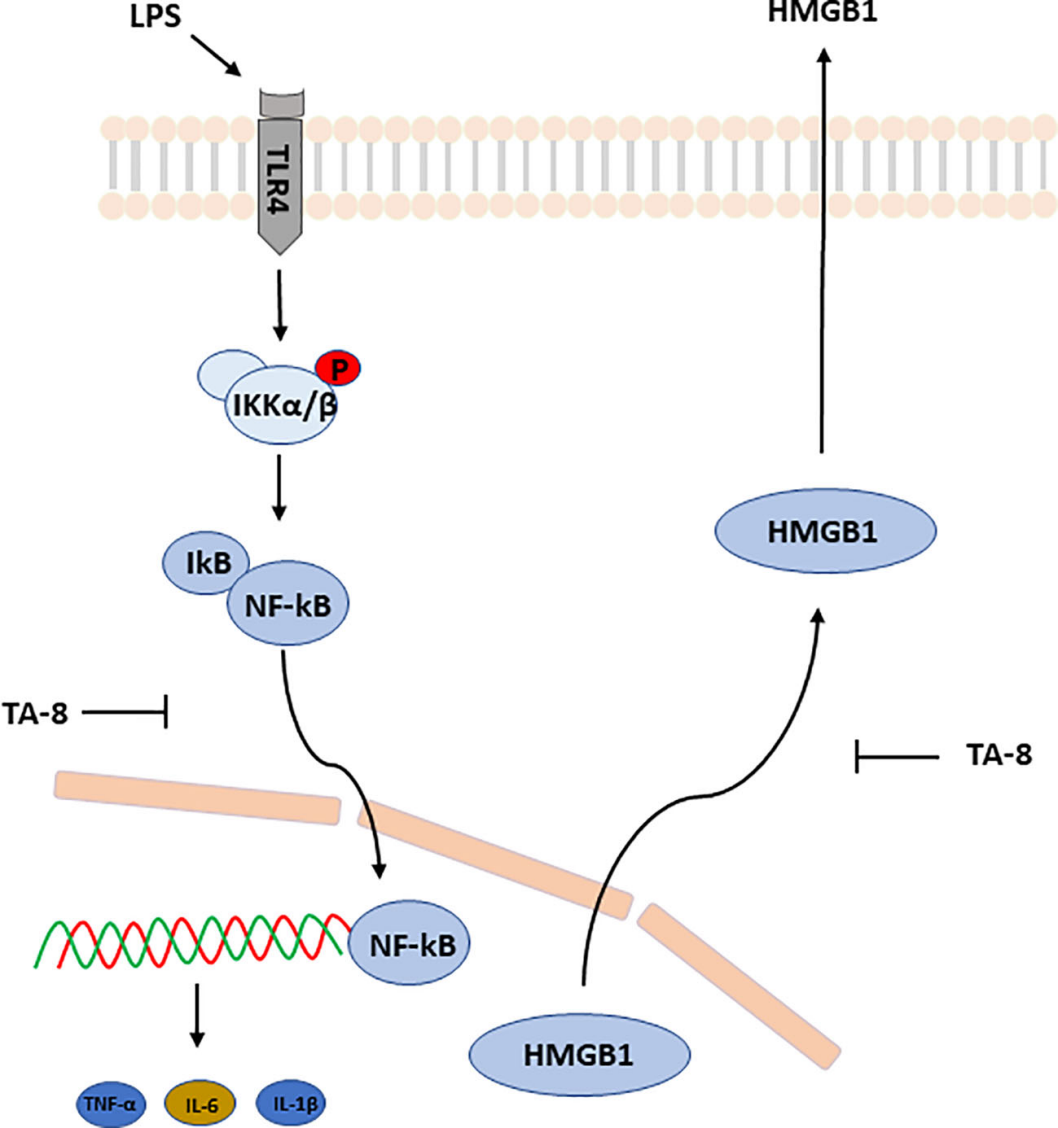
Schematic illustration of underlying mechanism of TA-8’s anti-inflammation activity. Toddalolactone (TA-8) protects lipopolysaccharide (LPS)-induced sepsis and attenuates LPS-induced inflammatory response by modulating HMGB1-NF-κB translocation.

Taken together, these results suggested that toddalolactone protects LPS-induced sepsis and attenuates LPS-induced inflammatory response by modulating HMGB1-NF-kB translocation (**Figure 7**).

## Discussion

Sepsis refers to the systemic inflammatory response syndrome (SIRS) caused by infection. In recent years, despite the rapid progress of anti-inflammatory therapy and multi-organ function support technology, the mortality rate of sepsis is still up to 30 to 70%. Inflammation plays a pivotal role in the pathological process of sepsis. Sepsis is usually treated by finding the primary lesion and using anti- inflammatory and antibacterial agents. Several therapeutic strategies for sepsis aim to modulate inflammatory responses such as low-dose glucocorticoids, C-reactive protein inhibitors, TNF-α and IL-1β receptor antagonists, recombinant IL-17, and PD-1 immunotherapy, etc. (Delano and Ward, 2016). Although these intervention strategies have some effect, it is still impossible to find an effective treatment to adapt to various complicated conditions. Therefore, researchers are still studying the role of different immune response mediators and looking for better ways to regulate the balance of immune responses.

There are three mouse models commonly used to mimic the pathophysiology of sepsis: endotoxin shock model induced by lipopolysaccharide (LPS), high-dose live bacterial infection model (generally *Escherichia coli*), and cecal ligation and perforation model. These three animal models have different types of infection sources and are unable to replicate the human sepsis process perfectly, but still can assess the contribution of key proteins as molecular targets in sepsis and explore the therapeutic effects of drugs on sepsis.

In this study, a mouse model of septic shock was induced by intraperitoneal injection of LPS. The effects of TA-8 on the secretion of inflammatory factors in the early, late stages of inflammation in septic mice were studied by ELISA and q-PCR. We found that TA-8 has a significant inhibitory effect on the secretion of inflammatory factors. Furthermore, the organ function of sepsis mice was evaluated. We found that TA-8 can improve serum liver function index in mice with sepsis during MODS. At the same time, the protective effects of TA-8 on lung, liver, and kidney of septic mice were evaluated by HE staining.

Nitric oxide (NO), which has the advantages of low cost and rapidity, is a common indicator for screening anti-inflammatory activities of drugs in the laboratory. NO is an important player in the host immune response and has a role in inflammation regulation and bactericidal action. It is a signaling molecule involved in the regulation of vascular hemodynamics, which regulates local blood flow by activating cGMP and mediates cell-cell interactions by regulating the expression of adhesion markers under various conditions (Thom et al., 2013; Chandrasekar et al., 2015). NO synthase (NOS) exists in three different subtypes and is expressed in different cell types, which leads to the functional diversity of NO in different cells. Yadav, S. et al. found that the production of inflammatory cytokines and chemokines decreased and the infiltration of neutrophils into the abdominal cavity was alleviated, which led to reduced overall inflammatory response in septic mice lacking inducible nitric oxide synthase (NOS2). However, at the same time, the bacterial burden in the abdominal cavity is aggravated, coupled with worsened organ damage and eventually leads to a decrease in the survival rate (Yadav et al., 2018). The specific role of NO in the pathophysiology of pre-clinical and clinical studies of sepsis is controversial (Lupp et al., 2013).

Although NO is a common inflammatory marker, considering the two-sidedness of NO on sepsis, it may not be appropriate to use NO as the evaluation standard for efficacy. Therefore, this experiment did not use it as an indicator of inflammatory factors for pharmacodynamic analysis.

Traditionally, the diagnosis of sepsis is based on the clinical signs and symptoms of sepsis, such as fever, tachycardia, and shortness of breath, and is tested by blood culture bacteria. Traditional diagnostic methods are time consuming and laborious, and thus biomarkers of sepsis that are more advantageous in diagnosis have been developed. The most commonly used biomarkers for identifying sepsis are lactic acid and C-reactive protein (CRP) (Sharma et al., 2018). Lactic acid and CRP are the most widely used markers to determine the severity of sepsis and its progression, but they are not specific markers of sepsis (Shabuj et al., 2017). Because of the non-specificity of these two indicators, we did not use them to evaluate the efficacy of TA-8. Procalcitonin (PCT) and interleukin-6 (IL-6) have become popular biomarkers for predicting the prognosis of sepsis in the last decade (Ruiz-Rodriguez et al., 2012; Jekarl et al., 2013; Mat-Nor and Md Ralib, 2014; Mat-Nor et al., 2016). PCT is a specific marker of sepsis and is elevated in pro-inflammatory stimuli, especially bacterial-derived immune responses, and is also strongly associated with the severity of systemic inflammation (Elbirt et al., 2001; Enguix et al., 2001). Procalcitonin is used clinically to guide the shortening of antibiotic treatments, while other biological indicators do not have this ability, which can be used to monitor the process of systemic inflammation (Harrison and Collins, 2015; Westwood et al., 2015). PCT has an irreplaceable status in the treatment of sepsis. The current application of PCT in the treatment of sepsis is mainly focused on its diagnostic value, however, its prognostic utility has conflicting results (Han et al., 2003; Meng et al., 2009; Bartolovic et al., 2011; Magrini et al., 2013; Bustos and Padilla, 2015). Parli, S. E. et al. reviewed the use of procalcitonin in patients with different trauma and postoperative acute care surgery. It is believed that procalcitonin helps identify infections. However, the concentration of PCT markers varies among patients with numerous infection complications. Therefore, there is currently no recommended standard PCT level evaluation method (Parli et al., 2018). We use PCT as one of the biomarkers of mouse sepsis and hope to play a positive role in the application of PCT.

HMGB1 is an important late inflammatory mediator, and its secretion has two mechanisms, passive cell mechanism and active cellular mechanism. The passive release of HMGB1 occurs instantaneously when cells are necrotic. Active HMGB1 secretion occurs 16 to 32 h after the onset of acute endotoxemia, and is secreted very late compared to the release of most other pro-inflammatory factors (Wang et al., 1999), so HMGB1 is both an early inflammatory factor and late mediators of inflammation. The active release of HMGB1 requires two key steps: HMGB1 is transported from the nucleus to the cytoplasm *via* the JAK-STAT1 signaling pathway, which depends on the key lysine acetylation of the two nuclear localization sites (Bonaldi et al., 2003; Lu et al., 2014). At the same time, the molecular modification of HMGB1 prevented the continuous two-way shuttle of HMGB1 between the cytoplasm and nucleus, and led to the accumulation of highly acetylated HMGB1 into the cytoplasm. The second step involves programmed inflammatory cell death where HMGB1 is transferred from the cytoplasm to the extracellular (Lamkanfi et al., 2010; Lu et al., 2013), or delivered extracellularly through exocytosis of the cell (Gardella et al., 2002). Inflammatory-induced release of HMGB1 is highly acetylated HMGB1, and this highly acetylated HMGB1 is not produced by cell death caused by necrosis or apoptosis. Thus, highly acetylated HMGB1 is a biomarker of inflammation.

We studied the effect of TA-8 on HMGB1 by LPS-induced RAW264.7 inflammatory model and intraperitoneal injection of LPS-induced mouse sepsis model. In addition, the redox state of HMGB1 released after heat shock is mainly in the form of disulfide bonds. Moreover, the redox state of HMGB1 released after heat shock is mainly in the form of disulfide bonds, which form disulfide after cell necrosis and sulfonyl HMGB1 after apoptosis. Heat shock pretreatment in mice can reduce mice mortality rate. In this experiment, the temperature should be controlled using air conditioning or heating to maintain indoor temperature, and avoid the use of small animal thermostats such as electric blankets.

Toll-like receptor 4 (TLR4) is a transmembrane receptor expressed in immune cells and vital organs. The TLR4 signaling pathway is activated and mediates the release of inflammatory mediators during sepsis. Zhou, et al. and Yang found that TLR4 knockout mice had lower mortality after cecal ligation and puncture models. At the same time, the expression levels of IL-1β, IL-6, TNF-α, and neutrophil infiltration were reduced (Zhou et al., 2018; Yang et al., 2018). The anti-inflammatory effect of inhibiting TLR4 activation is to block the classical NF-κB signaling pathway. Under normal conditions, NF-κB is present in the cytoplasm. When TLR4 is activated by LPS, it is phosphorylated into an active state and enters the nucleus. Its phosphorylation is regulated by IκB. IκB phosphorylation is a prerequisite for NF-κB activation and is mediated by the IKK complex. Sub ubiquitination then occurs and is degraded by the proteasome (Qureshi et al., 2003; Shen et al., 2006). The IKK complex consists of at least three subunits, including IKBKA and IKBKB and the regulatory subunit IKBKC. IKBKC, as part of the IKK complex, regulates IKBKB by linking the complex with upstream signaling molecules, and it also inhibits the activity of IKK complexes (Shifera, 2010).

We investigated the effect of TA-8 on the TLR4-IKBKB-IκBα-NF-κB signaling pathway using the LPS-induced RAW264.7 inflammatory model. We found that TA-8 reduced NF-κB transcriptional activity and inhibited its entry into the nucleus, while reducing LPS-induced IKBKB expression upregulation and IκBα phosphorylation.

Uncontrolled inflammatory immune response triggers up-regulation or down-regulation of protein expression in multiple pathways, involving cardiovascular, endocrine, neurological, metabolic, coagulation, energy metabolism, etc., which leads to tissue hypoxia, mitochondrial dysfunction, and cell death and subsequently leads to impaired organ function (Tidswell and Singer, 2018). The treatment of sepsis needs to inhibit the development of inflammation. Clinically, non-specific anti-inflammatory drugs are often used to treat sepsis, which can reduce the incidence of new organ failure and the 28-day mortality rate. As the traditional Chinese medicine, *T. asiatica* L. has a clear development potential. This study found the anti-inflammatory activity of *T. asiatica* L. active ingredient TA-8 and contributed to the development of modern innovative Chinese medicine.

## Data Availability Statement

The raw datasets used to support the findings of this study are available from the corresponding author on reasonable request.

## Ethics Statement

The animal study was reviewed and approved by TCM Animal Research Committee (TCM-LAEC2014005) of Tianjin University.

## Author Contributions

GF, JN and YZ designed experiments. YZ, JS, ZL, SF and JD carried out experiments. YZ and LL analyzed experimental results. JN wrote the manuscript. All authors reviewed and edited this manuscript.

## Funding

This study was funded by the Tianjin Outstanding Youth Science Foundation (No. 17JCJQJC46200), the National Natural Science Foundation of China (No.81774050), the Natural Science Foundation of Tianjin (17JCYBJC29000), the National Key Basic Research Program of China (973 Program) (No.2012CB518404) and scientific research project of Tianjin Education Commission (2019KJ072).

## Conflict of Interest

The authors declare that the research was conducted in the absence of any commercial or financial relationships that could be construed as a potential conflict of interest.
